# Supplementary material for: Splice-Junction-Based Mapping of Alternative Isoforms in the Human Proteome
Source: Cell Rep. Author manuscript; Available in PMC 2020 Jan 15. (PMC6961840; doi:10.1016/j.celrep.2019.11.026)
Supplement: 2 [file NIHMS1546469-supplement-2.zip › DF1/PXD009737/TestisTrypsin/TestisTrypsin_9_PHLDB1_VESLPAEPLPTDDPAGQQVIEEQR.pdf]

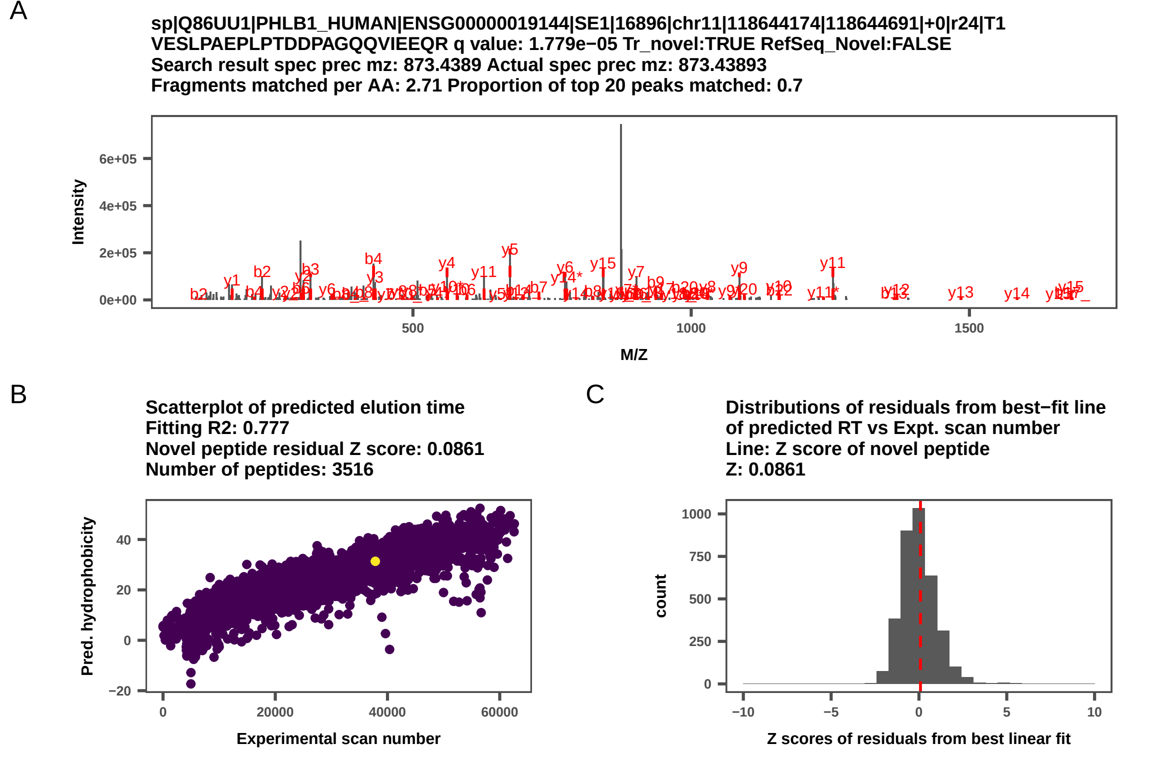
A
sp|Q86UU1|PHLB1_HUMAN|ENSG00000019144|SE1|16896|chr11|118644174|118644691|+0|r24|T1
VESLPAEPLPTDDPAGQQVIEEQR q value: 1.779e−05 Tr_novel:TRUE RefSeq_Novel:FALSE
Search result spec prec mz: 873.4389 Actual spec prec mz: 873.43893
Fragments matched per AA: 2.71 Proportion of top 20 peaks matched: 0.7
Intensity
6e+05
4e+05
2e+05
0e+00
500
1000
1500
M/Z
B
Scatterplot of predicted elution time
Fitting R2: 0.777
Novel peptide residual Z score: 0.0861
Number of peptides: 3516
Pred. hydrophobicity
40
20
0
−20
0
20000
40000
60000
Experimental scan number
C
Distributions of residuals from best−fit line
of predicted RT vs Expt. scan number
Line: Z score of novel peptide
Z: 0.0861
count
1000
750
500
250
0
−10
−5
0
5
10
Z scores of residuals from best linear fit
